# Supplementary material for: Gauging the happiness benefit of US urban parks through Twitter
Source: PLoS One. 2022 Mar 30;17(3):e0261056. doi: 10.1371/journal.pone.0261056 (PMC8967001; doi:10.1371/journal.pone.0261056)
Supplement: S1 Appendix — (ZIP) [file pone.0261056.s001.zip › si_030122.pdf]

# Appendix S1: Gauging the happiness benefit of US urban parks through Twitter

**Appendix A** Twitter's 'spritzer' streaming API offers a random selection of up to 1% of all messages, with specific linguistic or spatial filters enabling a higher percentage. For the present study, we collected messages tagged with GPS coordinates during the years 2012–2015. During this period, geolocated messages comprised roughly 1% of all messages. As a result, filtering on GPS enabled us to collect nearly 100% of all such messages.

**Appendix B** As is common in natural language processing, we define 'stop words' as individual words that we mask from sentiment analysis. These are words that we identify as frequent in our tweets, but that contribute neutral or context-dependent sentiment. We do not include the word *park* in our analysis. We removed the words *closed, traffic,* and *accident* because they frequently appeared in geo-located tweets from automated traffic posts. We removed words found in the names of the parks (e.g., *golden* and *gate*). Several cities had increased frequencies for the positive words *art, museums, gardens,* and *zoos* in their parks. Even though these words were not in the official park names, we removed them from our analysis. Several parks had the positive words *music* and *festival* appear frequently, so we removed these two words. For each city, we identified a list of stop words to remove by manually checking the 10 most influential words contributing to the difference between in-park and control tweets. Finally, we removed words that referred to a specific location (e.g., *beach*) or were being used in a significantly different way than they were originally rated for happiness (e.g. *ma* as shorthand for Massachusetts rather than mother) were removed (See Appendix Table 1).

Overall, the majority of words we masked were positive, with average happiness scores greater than 6 as seen in S1 Figure. As a result, we expect that the happiness benefit reported in our results is a lower bound.

**Appendix C** Tweets with any of the following hashtags were removed from our study sample: #jobs, #job, #getalljobs, #hiring, #tweetmyjobs, #careerarc, #hospitality, #healthcare, #nursing, #marketing, #sales, #clerical, and #it.

**Appendix D** In addition to the proximate time control described in the Methods section, we employed a secondary control to investigate the happiness benefit methodology. In this method, we selected a 'user control' tweet: a random message from the same user posted out-of-park. If an account's message history consisted entirely of in-park tweets, the account was removed from the sample as they were likely a tourist or business located adjacent to the park. The user control allows us to estimate a happiness benefit for the users during their park visits compared to tweets when they were not in the parks. We performed the same happiness benefit calculation for each of the 25 cities and include those results in S2 Figure. For our 'user control' group, the mean happiness benefit for the cities in our sample ranged from $-0.02$ to .05 (S2 Figure). We also plot the mean happiness benefit against park spending for capita and Park Score® in S3 Figure. The overall benefit reduction observed for the User Control, when compared with the time control, suggests that individuals who tweet from within parks generally use happier words than individuals who do not visit parks.

**Appendix E** We estimated the happiness benefit by hour of day across all cities (S4 Figure). While 8:00 and 9:00AM are slightly lower, the rest of the day's happiness benefit ranges overlap, showing that our other results are not biased by certain hours of the day (e.g., leaving the office).

**Appendix Table 1** Stop words selected for individual cities based on frequency analysis and contextual meaning.

| City | Stop Words |
|---|---|
| San Francisco | young, flowers |
| Phoenix | hospital |
| Jacksonville | science |
| Austin | limits |
| San Diego | sea |
| Washington | war, bill, united, health |
| Seattle | health, surgery, emergency |
| Chicago | riot |
| Houston | hospital, delay, stop, science |
| Cleveland | beach, island |
| Boston | ma, partners |
| New York | natural |
| San Antonio | cafe |
| Dallas | health |
| Philadelphia | independence |
| Los Angeles | science |
| San Jose | christmas, raging |
| Denver | nature, science, international |
| Memphis | steal, sugar |
| Charlotte | shot, young |
| Indianapolis | health |
| Columbus | roses |
