# Supplementary figures and images for: Gauging the happiness benefit of US urban parks through Twitter
Source: PLoS One. 2022 Mar 30;17(3):e0261056. doi: 10.1371/journal.pone.0261056 (PMC8967001; doi:10.1371/journal.pone.0261056)

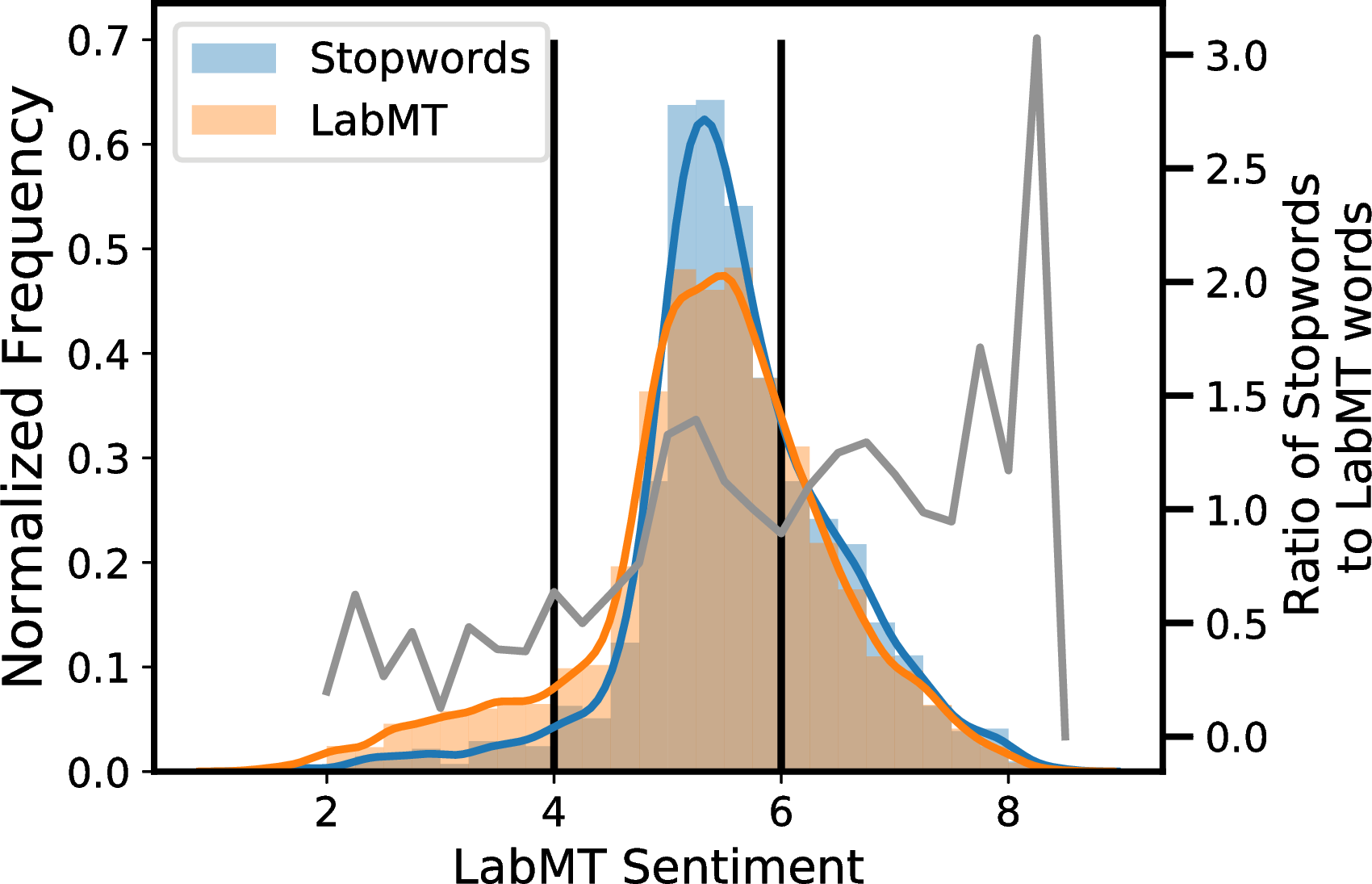

Supplement: S1 Fig — Our analysis is conservative as the ratio is higher for positive words (> 6) compared to negative words (< 4). Words between 4 and 6 are not included in our analysis. (TIF) [file pone.0261056.s002.tif]

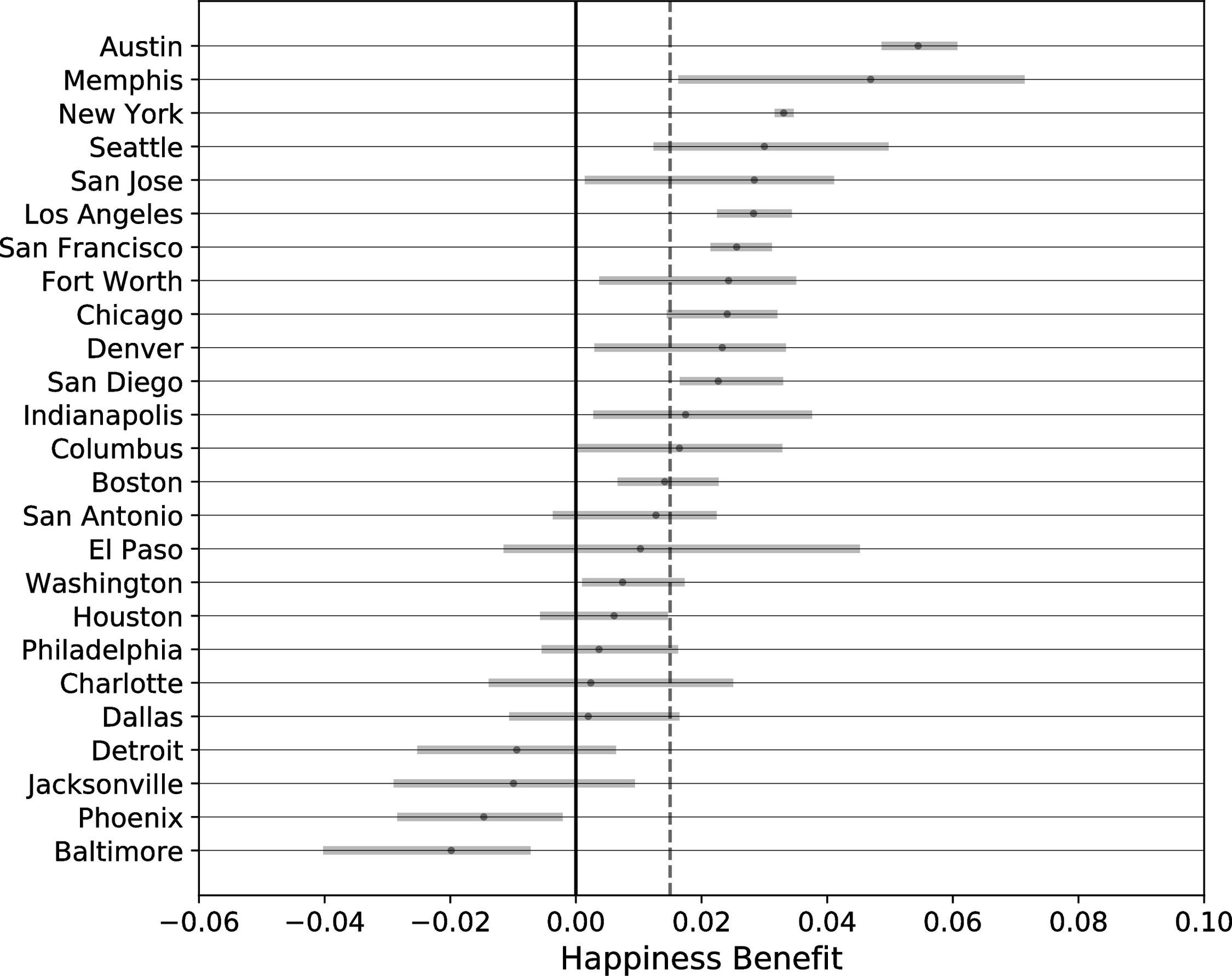

Supplement: S2 Fig — Happiness benefit by city. We derive each city’s full range of values from 10 bootstrap runs, for which we randomly selected 80% of tweets. Darker dots represent mean value from bootstrap runs. For each city, the control group consists of 1 random, non-park tweet from each user paired with an in-park tweet. (TIF) [file pone.0261056.s003.tif]

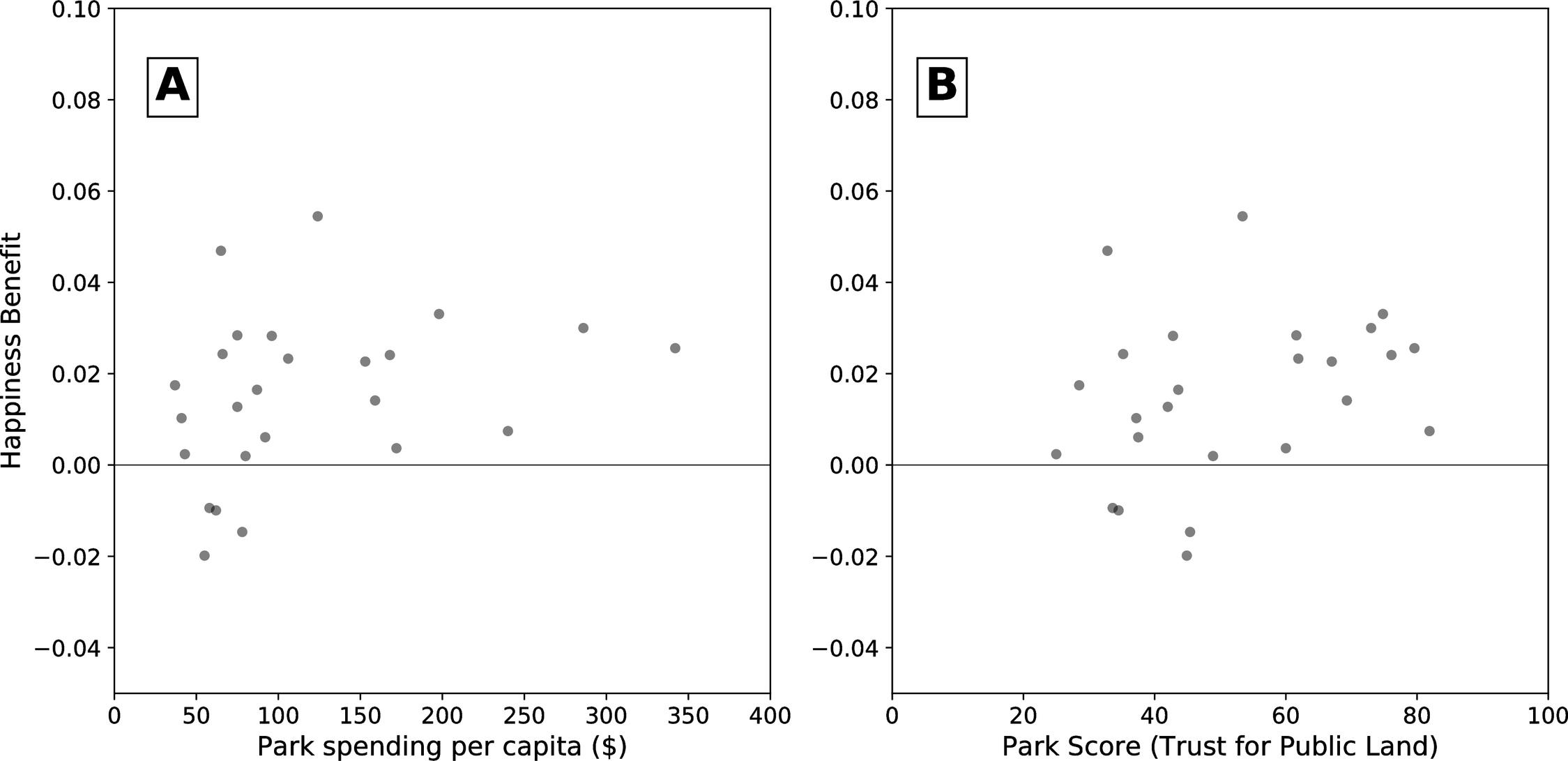

Supplement: S3 Fig — A. The left panel shows park spending per capita vs mean happiness benefit by city. Park spending per capita is from Trust for Public Land (TPL) data. B. The right panel shows ParkScore® vs mean happiness. The TPL calculates ParkScore® annually from measures of park acreage, access, investment, and amenities, and is scaled to a maximum score of 100. (TIF) [file pone.0261056.s004.tif]

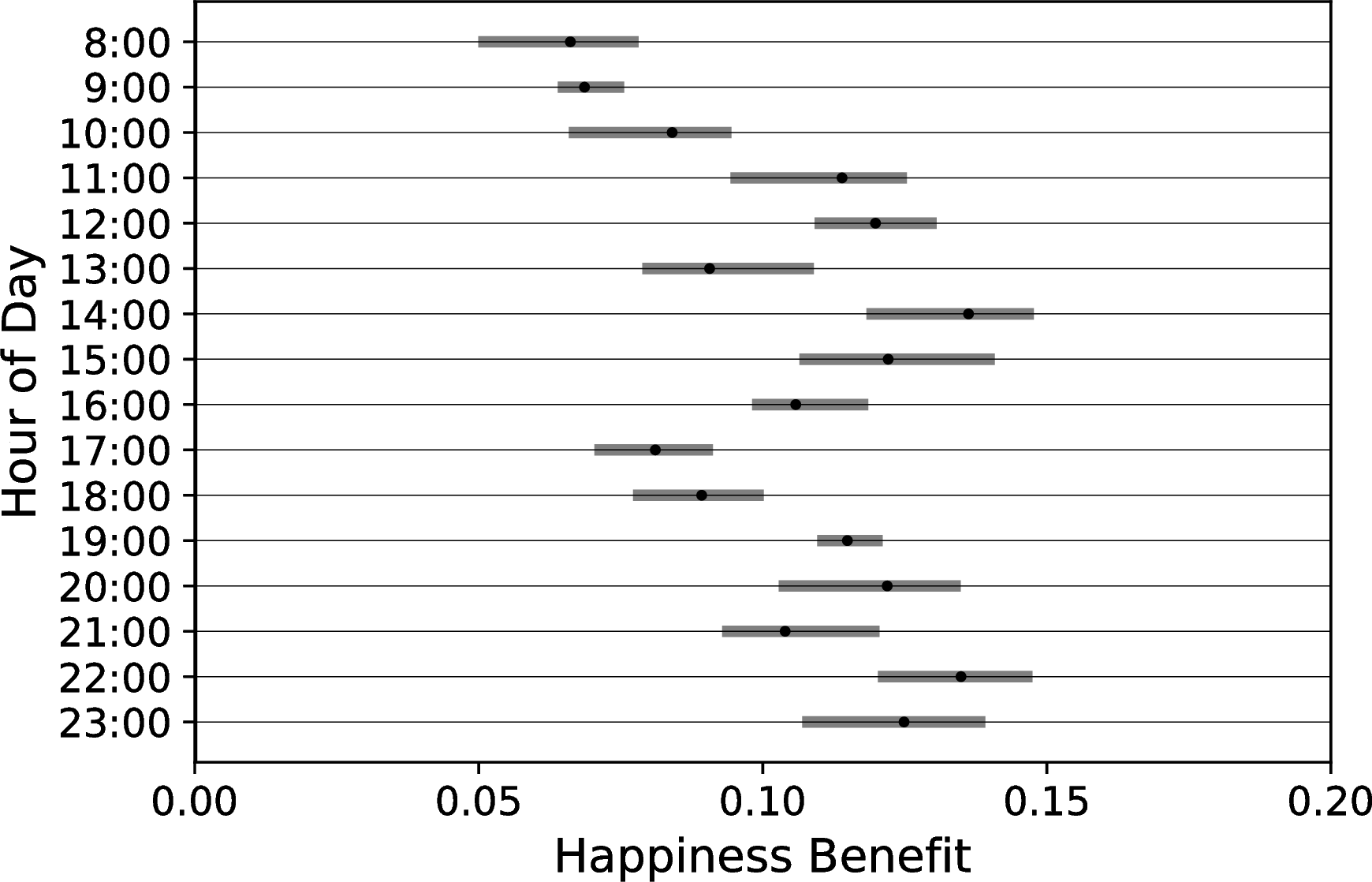

Supplement: S4 Fig — The range is the full range of happiness benefit estimates from 10 runs, sampling 80% of tweets. 1,000 random in-park tweets were pooled in each group from each city. Control tweets were selected as tweets most temporally proximate to the in-park tweet from the same city. (TIF) [file pone.0261056.s005.tif]
