# Supplementary material for: Gauging the happiness benefit of US urban parks through Twitter
Source: PLoS One. 2022 Mar 30;17(3):e0261056. doi: 10.1371/journal.pone.0261056 (PMC8967001; doi:10.1371/journal.pone.0261056)
Supplement: Attachment — Submitted filename: Response to Reviewers.docx [file pone.0261056.s006.docx]

Editors

*PLOS ONE*

May 21, 2021

Dear Dr. Chen:

We thank you and the reviewers for the constructive feedback and for the opportunity to revise and resubmit. We have addressed all comments and are pleased to submit our revised manuscript, *Gauging the happiness benefit of US urban parks through Twitter,* for inclusion in the *Urban Ecosystems* collection at *PLOS ONE.* We have attached our responses to both editor and reviewer comments below.

We have submitted a fully reformatted manuscript as well as a marked-up version produced with latexdiff showing revisions from the original submission.

We appreciate your consideration and look forward to your response.

Best regards,

Aaron J. Schwartz

[Aaron.J.Schwartz@colorado.edu](mailto:Aaron.J.Schwartz@colorado.edu)

Peter Dodds

Jarlath O’Neil-Dunne

Taylor Ricketts

Chris Danforth

*When submitting your revision, we need you to address these additional requirements.*

*1. Please ensure that your manuscript meets PLOS ONE's style requirements, including those for file naming. The PLOS ONE style templates can be found at*

[*https://journals.plos.org/plosone/s/file?id=wjVg/PLOSOne_formatting_sample_main_body.pdf*](https://journals.plos.org/plosone/s/file?id=wjVg/PLOSOne_formatting_sample_main_body.pdf) *and*

[*https://journals.plos.org/plosone/s/file?id=ba62/PLOSOne_formatting_sample_title_authors_affiliations.pdf*](https://journals.plos.org/plosone/s/file?id=ba62/PLOSOne_formatting_sample_title_authors_affiliations.pdf)

We have adapted PLOS ONE’s style requirements. We have included a version of the original manuscript submission (old_manuscript.pdf) in PLOS ONE format, the revised manuscript in PLOS ONE format manuscript.pdf) and a marked-up version of the manuscript produced with latexdiff (revised_manuscript_with_track_changes.pdf).

*2. Thank you for stating the following in the Acknowledgments Section of your manuscript:*

*"We are grateful for support from the NSF GRFP program, the Gund Institute for the Environment Catalyst Award program, and a gift from MassMutual Life Insurance Company."*

*We note that you have provided funding information that is not currently declared in your Funding Statement. However, funding information should not appear in the Acknowledgments section or other areas of your manuscript. We will only publish funding information present in the Funding Statement section of the online submission form.*

*Please remove any funding-related text from the manuscript and let us know how you would like to update your Funding Statement. Currently, your Funding Statement reads as follows:*

*"AJS is supported by NSF GRFP program DGE1451866.* [*https://www.nsfgrfp.org/*](https://www.nsfgrfp.org/)

*All authors are supported by Gund Institute for the Environment Catalyst Award program.* [*https://www.uvm.edu/gund/gund-catalyst-awards*](https://www.uvm.edu/gund/gund-catalyst-awards)

*The funders had no role in study design, data collection and analysis, decision to publish, or preparation of the manuscript."*

*Additionally, because some of your funding information pertains to commercial funding, we ask you to provide an updated Competing Interests statement, declaring all sources of commercial funding.*

Here is an updated Funding Statement:

AJS is supported by NSF GRFP program DGE1451866. https://www.nsfgrfp.org/

All authors are supported by Gund Institute for the Environment Catalyst Award

program. <https://www.uvm.edu/gund/gund-catalyst-awards>

Authors associated with the MassMutual Center for Excellence for Complex Systems and Data Science are supported in part by a gift from MassMutual Life Insurance Company.

The funders had no role in study design, data collection and analysis, decision to

publish, or preparation of the manuscript.

*In your Competing Interests statement, please confirm that your commercial funding does not alter your adherence to PLOS ONE Editorial policies and criteria by including the following statement: "This does not alter our adherence to PLOS ONE policies on sharing data and materials.” as detailed online in our guide for authors*  [*http://journals.plos.org/plosone/s/competing-interests*](about:blank)*.  If this statement is not true and your adherence to PLOS policies on sharing data and materials is altered, please explain how.*

*Please include the updated Competing Interests Statement and Funding Statement in your cover letter. We will change the online submission form on your behalf.*

Updated Competing Interests Statement:

Authors associated with the MassMutual Center for Excellence for Complex Systems and Data Science are supported in part by a gift from MassMutual Life Insurance Company. This does not alter our adherence to PLOS ONE policies on sharing data and materials.

*3. We note that you have stated that you will provide repository information for your data at acceptance. Should your manuscript be accepted for publication, we will hold it until you provide the relevant accession numbers or DOIs necessary to access your data. If you wish to make changes to your Data Availability statement, please describe these changes in your cover letter and we will update your Data Availability statement to reflect the information you provide.*

Data will be made available upon acceptance at the following DOI: 10.6084/m9.figshare.12915329

*4. We note that Figure 4 in your submission contains map images which may be copyrighted. All PLOS content is published under the Creative Commons Attribution License (CC BY 4.0), which means that the manuscript, images, and Supporting Information files will be freely available online, and any third party is permitted to access, download, copy, distribute, and use these materials in any way, even commercially, with proper attribution. For these reasons, we cannot publish previously copyrighted maps or satellite images created using proprietary data, such as Google software (Google Maps, Street View, and Earth). For more information, see our copyright guidelines:* [*http://journals.plos.org/plosone/s/licenses-and-copyright*](http://journals.plos.org/plosone/s/licenses-and-copyright)*.*

*We require you to either (1) present written permission from the copyright holder to publish these figures specifically under the CC BY 4.0 license, or (2) remove the figures from your submission:*

*(1) You may seek permission from the original copyright holder of Figure 4 to publish the content specifically under the CC BY 4.0 license.*

*We recommend that you contact the original copyright holder with the Content Permission Form (*[*http://journals.plos.org/plosone/s/file?id=7c09/content-permission-form.pdf*](about:blank)*) and the following text:*

*“I request permission for the open-access journal PLOS ONE to publish XXX under the Creative Commons Attribution License (CCAL) CC BY 4.0 (*[*http://creativecommons.org/licenses/by/4.0/*](about:blank)*). Please be aware that this license allows unrestricted use and distribution, even commercially, by third parties. Please reply and provide explicit written permission to publish XXX under a CC BY license and complete the attached form.”*

*Please upload the completed Content Permission Form or other proof of granted permissions as an "Other" file with your submission.*

*In the figure caption of the copyrighted figure, please include the following text: “Reprinted from [ref] under a CC BY license, with permission from [name of publisher], original copyright [original copyright year].”*

*(2) If you are unable to obtain permission from the original copyright holder to publish these figures under the CC BY 4.0 license or if the copyright holder’s requirements are incompatible with the CC BY 4.0 license, please either i) remove the figure or ii) supply a replacement figure that complies with the CC BY 4.0 license. Please check copyright information on all replacement figures and update the figure caption with source information. If applicable, please specify in the figure caption text when a figure is similar but not identical to the original image and is therefore for illustrative purposes only.*

*The following resources for replacing copyrighted map figures may be helpful:*

*USGS National Map Viewer (public domain):* [*http://viewer.nationalmap.gov/viewer/*](about:blank)

*The Gateway to Astronaut Photography of Earth (public domain):* [*http://eol.jsc.nasa.gov/sseop/clickmap/*](about:blank)

*Maps at the CIA (public domain):* [*https://www.cia.gov/library/publications/the-world-factbook/index.html*](about:blank) *and* [*https://www.cia.gov/library/publications/cia-maps-publications/index.html*](about:blank)

*NASA Earth Observatory (public domain):* [*http://earthobservatory.nasa.gov/*](about:blank)

*Landsat:* [*http://landsat.visibleearth.nasa.gov/*](http://landsat.visibleearth.nasa.gov/)

*USGS EROS (Earth Resources Observatory and Science (EROS) Center) (public domain):* [*http://eros.usgs.gov/#*](about:blank)

*Natural Earth (public domain):* [*http://www.naturalearthdata.com/*](about:blank)

We have decided to remove the figure at this time to avoid copyright issues.

Response to reviewers:

*Reviewer #1: The analysis in this article is very innovative and meaningful, it combines the Twitter platform and big data technology with the relationship between language and emotion, to explore the impact of urban parks and happiness benefits. However, several aspects need to be further explored.*

*1. The content of the literature review is not sufficient enough, and the paper does not accurately point out what previous studies have been done, and which aspects need to be filled in.*

Response: We have expanded our literature review and reorganized our introduction section (Lines 2-91). We now discuss relevant literature from four different types of approaches to studying nature contact (experimental, epidemiological, experience-based, social media) and explain the contributions of each to the field. We have more clearly explained what prior studies have done when examining urban parks and happiness, as well as the most relevant gaps.

*2. The titles of the figures are too long, adding the explanations of the figures in the text or adding some legends to the figure is more appropriate*.

Response: We have shortened the figure titles (See Figures 1-5). We have maintained detailed captions to ensure readers can fully parse the figures.

*3. More methods can be considered in the article to determine the relationship between green space and happiness in different geographical locations, such as geographically weighted regression.*

Our sample of 25 cities does not lend itself to regression methods. We are not trying to estimate a causal relationship between greenspace and happiness across locations. Instead, we have performed a variety of controls on our sentiment analysis – matching Tweets by time of day within the same city and user (Materials & Methods, S2. Appendix), removing a variety of bots and hashtags (S3. Appendix) and performing the bootstrap procedure in our estimate of happiness benefit. These methods help us compare and explore the relative mental benefit of urban park visitation across cities.

*Reviewer #2: This study estimated a happiness benefit, the difference in expressed happiness between in- and out-of-park tweets, for 25 cities in the US. The topic of this paper is worthwhile to be explored. However, the analysis is too simple to make the conclusions.*

*(1) This manuscript lacks necessary theoretical thinking.*

Thank you for your suggestions. We have added theoretical grounding to the introduction (Lines 19-47)

*(2）The literature review is too simple.*

We have significantly broadened the literature review, organized into 4 paragraphs (Lines 9-47)

*(3) There is little introduction and comment on the results of the analysis.*

We integrated our results and discussion sections into a single section, which we introduce on lines 186-189. We comment on the results of the analysis throughout this section (Lines 197-286).

Reviewer #3: The paper is an interesting analysis of social media data. I suggest several minor revisions:

*page 2: 'These pathways have been explored using a dose-response framework which describe the duration, frequency, and intensity of nature contact.' should be cited.*

Thank you for catching this omission. During our revision of the introduction and theoretical framework we removed this sentence. The original citation should have been:

Shanahan, D. F., Fuller, R. A., Bush, R., Lin, B. B., & Gaston, K. J. (2015). The Health Benefits of Urban Nature: How Much Do We Need? BioScience, 65(5), 476–485. https://doi.org/10.1093/biosci/biv032

*page 2 etc: Please call out the hypotheses more directly, using bold text or H1: xxx, so the reader is able to quickly access these important statements.*

Thank you, we have added 5 explicit hypotheses bolded and marked as H1-H4 on lines 55,60, 65, 69, 79. We now reference these in our results as well (see lines 192, 238-239, 252, 270).

*page 3, The first full paragraph seems to support another hypothesis.*

We added an additional explicit hypothesis on line 60 (H2B).

*page 4: Sentiment analysis - provide a sentence that explains the derivation of word scores as this information is fundamental to the paper's premise. Sentence at end of same paragraph is unclear.*

Thank you for this comment. We have edited the paragraph on Lines 119-133 to provide additional detail on the derivation of word scores and clarified the last sentence of the paragraph.

*Concluding Remarks: You may wish to address these observations and questions:*

*This is another study providing correlational connections between nature and health. Could there be a more refined methodology using social media that would reveal causal mechanisms?*

This is a key challenge in non-experimental approaches to studying nature contact and well-being. There are many determinants of mental health and it may be impossible to completely isolate the contribution of nature to someone’s well-being. We added additional detail to our introduction on the pros and cons of different methodological approaches (Lines 20-47) to clarify the utility of social media-based approaches and how they can contribute to collective efforts in studying nature and health. We attempt to address some of the correlational issues by including control groups for our in-park tweets, which we discuss in Materials & Methods (Lines 134-145) and expand upon in the Appendix (Lines 465-478).

*As I read I found myself thinking, what would be the happiness tweet scores for people in a museum, or at an event in a sports stadium, or even with friends at a bar? What would be happiness quotients comparisons? And considering that there would be similarities what would be the cost/benefit ratio of investing in different facilities to promote happiness?*

Thank you, this is a great suggestion. We now include a call for future research of this type in our Future Directions section (Lines 294-300). We note that there would be interesting methodological challenges to overcome such as data density (museums) or in filtering out stop words (sporting events) that should be informed by domain specific knowledge.
