# Supplementary material for: Gauging the happiness benefit of US urban parks through Twitter
Source: PLoS One. 2022 Mar 30;17(3):e0261056. doi: 10.1371/journal.pone.0261056 (PMC8967001; doi:10.1371/journal.pone.0261056)
Supplement: Attachment — Submitted filename: Response to Reviewer.docx [file pone.0261056.s007.docx]

*Reviewer #4: Based on the sentiment trend analysis from Twitter data, this paper studies the happiness scores in and outside parks in 25 cities in the United States, then calculates the happiness benefits. It’s a carefully done study and the results are of considerable interest, but there are still some issues to be clarified as follows.*

*(1) This paper uses the weighted average method to calculate the happiness benefits, which often conceals the high and low values in the areas outside the park. Is it possible to use thermograms to show the spatial distribution of annual happiness within these cities?*

Thank you for this thoughtful comment. While the weighted average may conceal high and low values, our method of analysis purposefully does this to ask a specific question about the relative effect of park visitation on happiness as compared to other urban environments. While producing location-specific thermograms is beyond the scope of this paper, we have acknowledged the potential obscuring of extreme values in the manuscript on lines 210-212.

“Prior work has shown that tweet happiness can vary within a city (even down to the

neighborhood level) and extreme sentiment values may be obscured by our weighted averaging procedure \cite{gibbons2019twitter}. We chose an aggregated approach to detect an overall signal about the effect of parks on happiness, but understanding detailed spatial patterns in happiness is an important future research direction.”

*(2) In the chapter of Temporal Analysis, the discussion of related results was missed. For example, the results in summer are higher than winter, and in weekends are higher than working days. It is worth considering that is this related to people’s travel habits.*

This is a helpful suggestion. We have added several sentence and an additional citation further expanding the discussion of our temporal analysis on lines 285-297.

“Possible interpretations of seasonal differences may include that warmer or sunnier weather in the summer leads to an increased benefit from park visitation. People may engage in longer visits to parks during summer months, engage in physical activity, or connect with friends during the summer, all of which may increase the benefits of spending time in a park \cite{Pretty2017}. Alternatively, more non-residents may be tweeting from parks during the summer, leading to greater within-park sentiment scores. Similar dynamics may be driving the higher happiness benefits on the weekend compared to weekdays, though all days of the week exhibited positive values (See Fig.~\ref{fig_bins}). Prior work has shown that people on Twitter are happiest on the weekends and during times of year with more daylight \cite{golder2011diurnal}. Nevertheless, our comparisons indicate that a sentiment benefit occurs throughout the day, week, and year, indicating that the effect is not purely driven by temporal patterns.”

*(3) We are happy to see your great results, but there is a lack of discussion at the end of this paper about the limitations, which is a common part of big data research. For example, the data comes from younger people who are more likely to share Twitter, and whether the higher spatial scale of the park (Fig4. A) is related to the surrounding population density and age structure is worth further discussion.*

Thank you, we have expanded our discussion and added a paragraph on the limitations of Twitter data around demographic representation. We have also included text discussing the limitations of our specifc methodology. For instance, we acknowledge that there are some confounding factors and potential biases in our approach of pooling tweets around specific types of parks. Please find these additions on lines 303-314.

“We acknowledge that studying human behavior using Twitter data involves several potential sources of bias. Active users on Twitter tend to be younger and more affluent than the population at large \cite{blank2017digital}. Instead of investigating how individual users and demographic sub-groups respond to nature contact, we attempt to estimate the aggregate effect of park visitation on happiness across a city. While our happiness benefit calculation uses same-city tweets as a control, the results may not generalize beyond Twitter users. We only use English language tweets which may limit our ability to generalize to other languages and cultures. We do not control for nearby demographics when assessing the happiness benefit of specific parks. For example, larger parks may be promixal to more affluent neighborhoods or associated with adjacent neighborhood age structure. While this may introduce bias across parks within cities, it should not impact our results comparing the total happiness benefit across cities.”
